# Evaluation of the Frequency of Anatomic Variations of the Paranasal Sinus Region by Using Multidetector Computed Tomography: A Hospital‐Based Cross‐Sectional Study

**DOI:** 10.1002/hsr2.70535

**Published:** 2025-03-05

**Authors:** Babar Ali, Zonaina Nadeem, Muhammad Naeem, Kinza Arif, Asad Zaman, Aqsa Noor, Muhammad Hassaan Zahid, Rimsha Mustafa, Aymar Akilimali, Jacob Leonard Ago

**Affiliations:** ^1^ University Institute of Radiological Sciences and Medical Imaging Technology, University of Lahore Lahore Pakistan; ^2^ Department of Research Medical Research Circle (MedReC) Bukavu DR Congo; ^3^ RMIT University Bundoora Australia

**Keywords:** functional endoscopic sinus surgery, multidetector computed tomography, paranasal sinuses

## Abstract

**Background and Aims:**

The sinus anatomy should be well‐understood by the sinus surgeons to carry out functional endoscopic sinus surgery carefully. That's why CT scans are vital to provide essential clarity and accuracy for comprehensive presurgical planning with minimal risks. The aim of this study was to evaluate the frequency of anatomic variations of the paranasal sinus region by using multidetector computed tomography.

**Methods:**

A cross‐sectional study of one hundred and fifty‐three patients of all age groups was carried out in the radiology department of Shalamar Hospital Lahore from 20 January, 2024 to 10 April, 2024 to evaluate anatomical variations presented to the facility by using multidetector CT. Data were collected using a standardized data collection sheet and analyzed using SPSS version 25.0.

**Results:**

The study included 94 males and 59 females with a median age of 43.9 years. Among the study population, 4.6% had aplasia of the frontal sinus, 84.3% had bilateral frontal sinus, but 13.7% had unilateral, 42.5% had more than 2 chambers of frontal sinus, and 9.8% had hypoplasia with persistent metopic suture. Four patterns of sphenoid sinus pneumatization were recognized: the conchal, the presellar, the sellar, and the post‐sellar, with prevalence rates of 2.0%, 11.1%, 28.1%, and 56.9%, respectively. 0.7% had hypoplasia of maxillary sinus, 9.2% had exostoses, 13.7% had extension of teeth roots to maxillary sinus, and 3.3% had maxillary sinus septations. Ethmoidal bulla, Agger nasi cells, and Haller cells had frequencies of 49%, 32.7%, and 30.7%, respectively.

**Conclusions:**

The most common anatomical variations are bilateral frontal sinus, post‐sellar pneumatization of sphenoid sinus, ethmoidal bulla in ethmoid sinus, and extension of teeth roots to maxillary sinus. Such characteristics and findings are to be evaluated for the management of pathologies associated with these variations and consequent surgical interventions.

## 1 Introduction

The four paranasal sinuses, which are paired structures in the center of the nasal cavity, are air‐filled chambers within the skull and facial bones [1]. At 25–28 weeks of gestation, a series of air‐filled chambers called the paranasal sinuses begin to form from the primitive choana [2]. The names of these sinuses, which include the frontal sinus, maxillary sinus, ethmoid sinus, and sphenoid sinus, correspond to the facial bone in which they are located.

The frontal sinuses are funnel‐shaped organs that are situated in the forehead's superior frontal bone, above the eyes. There is a primary septum that divides the left and right sides, but there may also be several septations [3]. It might not exist in certain people [4]. The maxillary sinus is also known as the Highmore Antrum. These sinuses can be found in the maxillary bone, underneath the eyes. The maxillary sinus, one of the four paranasal sinuses, is regarded as the biggest. The sinus has a form that is somewhat similar to a pyramid [5]. The ethmoid sinus, because of its complex anatomy, its honeycombed appearance, and the presence of delicate pathways, is called the ethmoid labyrinth. It is located in the anterior skull base between the nose and eyes. The most common pediatric infections mostly occur in this sinus. The shape of the sinuses is more like pyramids and is divided by the thin septa. The ethmoid bulla is the largest anterior ethmoidal air cell. Another common anatomical variation is the presence of Agger nasi cells [6]. Haller cells, which belong to the anterior group of ethmoidal air cells and are situated on the medial orbital floor, are also known as infraorbital ethmoid cells because of their location [7]. The sphenoid sinuses, which originate in the sphenoid bone, are situated behind the ethmoid sinuses [8]. Regarding the Sella turcica, there are four types of sphenoid sinus pneumatization. The sphenoid sinus, which looks like a solid block of bone without any air gaps below the sella turcica, has no or very little pneumatization in the conchal type [9].

The anatomical variations in these sinuses can lead to intracranial and infraorbital complications during functional endoscopic sinus surgery (FESS) [10], eliminating tissue that is blocking the osteometal complex (OMC) and allowing drainage not disturbing the normal anatomy of sinuses, which is named FESS. With the help of a fiberoptic nasal telescope, we can see the visualization of OMC, which gives us access to perform surgery on the specified areas. A tiny camera is attached to the eyepiece of the endoscope that provides us the complete image/video of the area on the monitor. Saving normal tissue, the abnormal tissue is ablated by microdebriders. From previous years, FESS can be used as the secure and productive surgery for paranasal sinus pathologies [11]. Analysis of these variations in Paranasal computed tomography (CT) is essential for preoperative assessment before functional endoscopic sinus surgery to avoid complications [12]. In terms of planning for FESS, multidetector computed tomography (MDCT) has also been proven to be superior to magnetic resonance (MR) imaging [13].

For radiologists, complete understanding of the anatomy of paranasal sinuses (PNS) is necessary. In the period of FESS, the surgeon should have a firm grip on the detection of anatomy, variations, and all the pathologies before the operation to steer clear of further problems. For the detection of paranasal sinuses, there are many imaging modalities that exist. Conventional radiography is very helpful in assessing the details of the frontal sinus and maxillary sinus. But it has the least part in the examination of the ethmoid sinus, sphenoid sinus, and nasal cavity [14]. Conventional radiography also gives less information about soft tissue areas of the sinuses [15].

Multi‐detector Computed tomography is a particular approach that displays slice images of the patient's body in a transaxial plane. It gives complete detail of anatomical variants and normal anatomy; it provides information on both bones and soft tissues around the PNS, which makes it the standard choice in PNS examination. It also talks about the spread of pathology inside and around the paranasal sinus. Any CT photograph is reconstructed in different planes by specific algorithms used in these techniques. The images are acquired by employing straight, continuous solid‐state sensors with an x‐ray source around the patient. It gives appealing bone features with a fine soft tissue survey. Before endoscopic surgeries, detection of major anatomical landmarks with their identifiable alternatives, this modality is the worthy course of action. The paranasal sinuses can be examined using computed tomography (CT) imaging to determine the existence of substantial anatomic abnormalities, the location and severity of the illness, and the precise position of the blockage [16]. MDCT is an improved version of the usual CT scan, which can be used to analyze body structures with more details and comparatively higher spatial resolution. MDCT is also used for accurate confirmations in case of any PNS disease [17]. The diagnosis of anatomical variations and sinonasal diseases through the use of a computed tomography (CT) scan provides more accurate guidance for clinical, therapeutic, and surgical options. The axial and coronal images provided by spiral CT make it easier to understand the size and relationship of the paranasal sinuses. The imaging technique of choice for evaluating paranasal sinuses and adjacent structures is currently the gold standard for research [18].

Besides CT, MRI has a smaller extent to supply the best images of the ostiometal area with other paranasal and nasal anatomical structures to the otolaryngologists and radiologists. In the MRI modality, soft tissue pathologies and lesions of the paranasal sinus are specifically examined. The main restriction of MRI is its failure to demonstrate the bony skeleton's anatomy in contrast to CT. That's why CT is currently the perfect modality of choice for the assessment of paranasal sinuses and its related structures [19]. In this study, we evaluated the frequency of anatomic variations of the paranasal sinus region by using MDCT.

## 2 Materials and Methods

A cross‐sectional descriptive study was conducted in the radiology department of Shalamar Hospital, Lahore, Punjab, Pakistan, from 20 January, 2024 to 10 April, 2024. The study included 153 patients of all age groups by using convenient sampling techniques. In this study, we included both genders of all ages and all those visiting for CT examination of paranasal sinuses. The equipment used was the SIEMENS 64‐slice CT machine.

Data were collected using a standardized data collection sheet. It was collected according to the variables of age, gender, and clinical history. Patients were scanned in a supine position. Angulation was parallel to the hard palate for the axial section and perpendicular to the hard palate for the coronal section. Images were taken from the axial, coronal, and sagittal planes. The CT scan was performed using the following parameters: a slice thickness of 1 mm, a tube voltage of 120 kilovolts (kV), and a tube current of 130 milliamperes (mA), with an exposure time of 1.5 s. Data was collected during the allocated period. These settings provided optimal image quality while minimizing radiation exposure, striking a balance between diagnostic accuracy and patient safety.

Written informed consent was taken from all the participants. All information and data collection were kept confidential. Ethical approval for this study **(Ethical Committee Ref No: REC‐UOL‐189‐01‐2024)** was provided by the Ethical Committee of the Research Ethics Committee, Faculty of Allied Health Sciences, The University of Lahore, on January 15th, 2024.

Statistical package SPSS version 25.0 was used for the evaluation of the data and compilation of results. Then the results were summarized in the form of tables. Quantitative data was explained in the form of frequency, mean, and standard deviation.

## 3 Results

Table 1 shows that the minimum age is 7, the maximum is 82, and the range is 75, whereas the mean ± standard deviation is 46.9 ± 17.7.

**Table 1:** Descriptive Statistics.

Parameter	Sample size (*N*)	Range	Minimum	Maximum	Mean	Std. Deviation
Age	153	75.0	7.0	82.0	44.0	17.7

**Figure 1:**
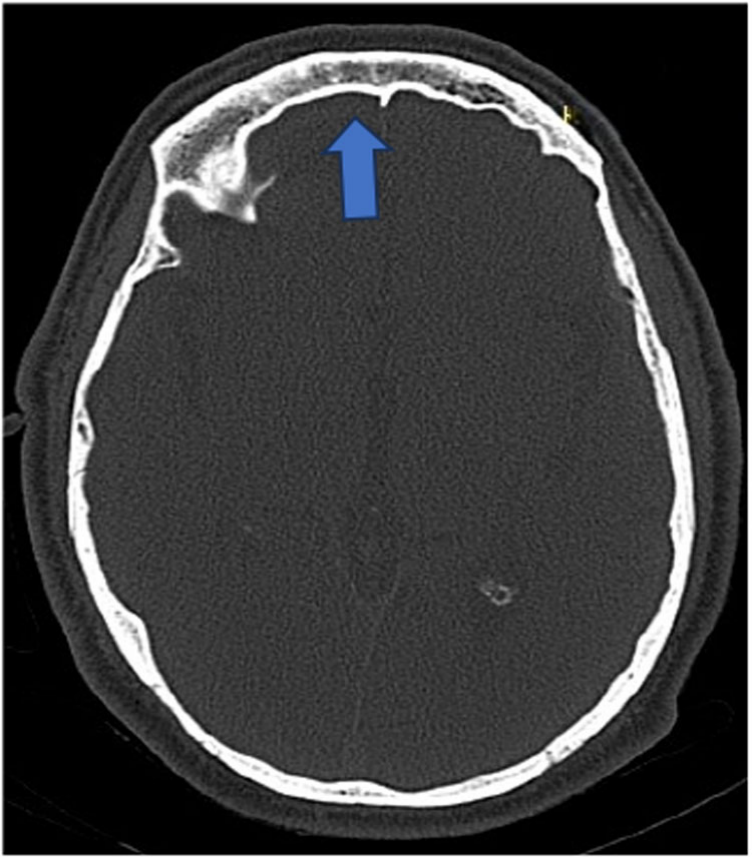
CT axial image shows aplasia of frontal sinus.

**Figure 2:**
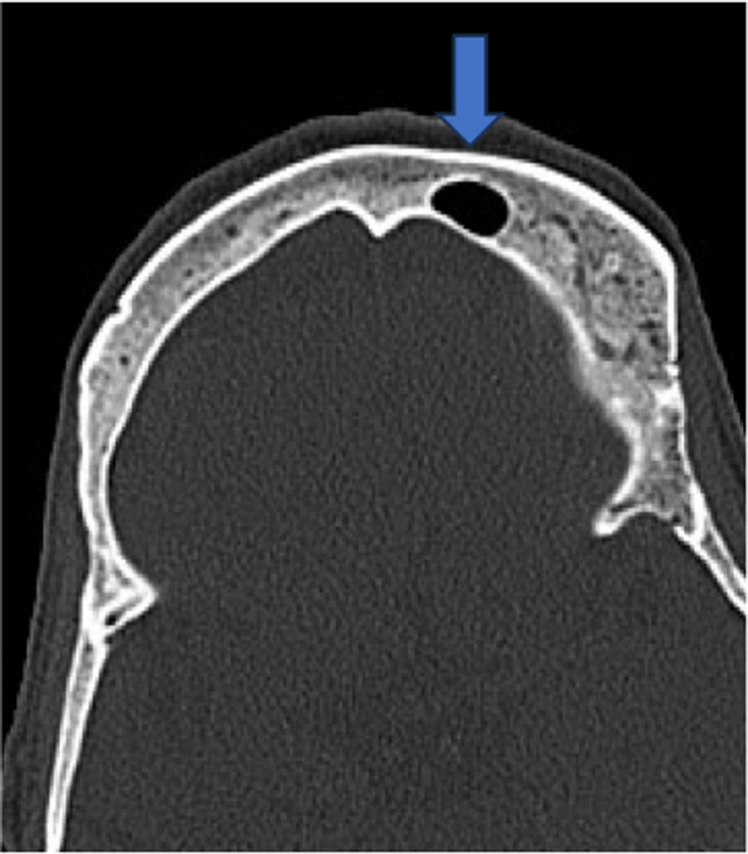
CT axial image shows unilateral frontal sinus.

**Figure 3:**
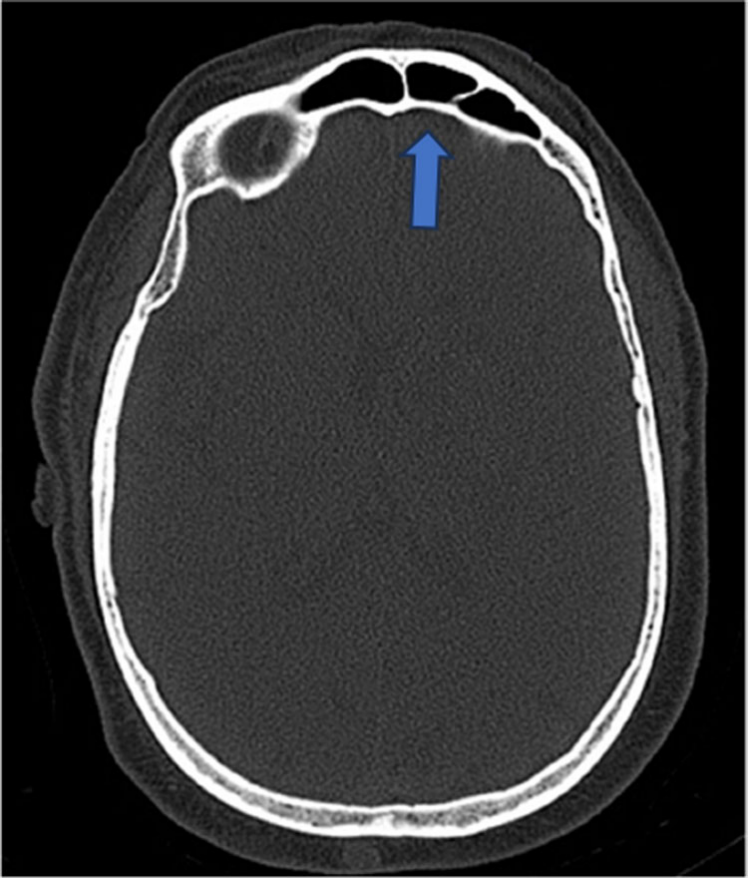
CT axial image shows more than two chambers of frontal sinus.

Table 2 shows that out of 153, *n* = 146 (95.4%) have no aplasia of the frontal sinus, while *n* = 7 (4.6%) patients have aplasia of the frontal sinus (Figure 1), *n* = 129 (84.3%) have bilateral frontal sinus, but *n* = 3 (2.0%) have no frontal sinus, *n* = 21 (13.7) have unilateral frontal sinus (Figure 2), *n* = 88 (57.5%) have no more than two chambers, but *n* = 65 (42.5%) have more than two chambers (Figure 3), *n* = 138 (90.2%) have no hypoplasia with persistent metopic suture, while *n* = 15 (9.8%) have hypoplasia with persistent metopic suture.

**Table 2:** Anatomical variations of frontal sinus.

Sinus type	Normal variations	Occurance	*N*
Frontal sinus	Aplasia	Present 7 (4.6%)	**153**
		Absent 146 (95.4%)	
	Unilateral/Bilateral	Unilateral 21 (13.7%)	
		Bilateral 129 (84.3%)	
		Absent 3 (2.0%)	
	More than 2 chambers	Present 65 (42.5%)	
		Absent 88 (57.5%)	
	Hypoplasia with persistent metopic suture	Present 15 (9.8%)	
		Absent 138 (90.2%)	

**Figure 4:**
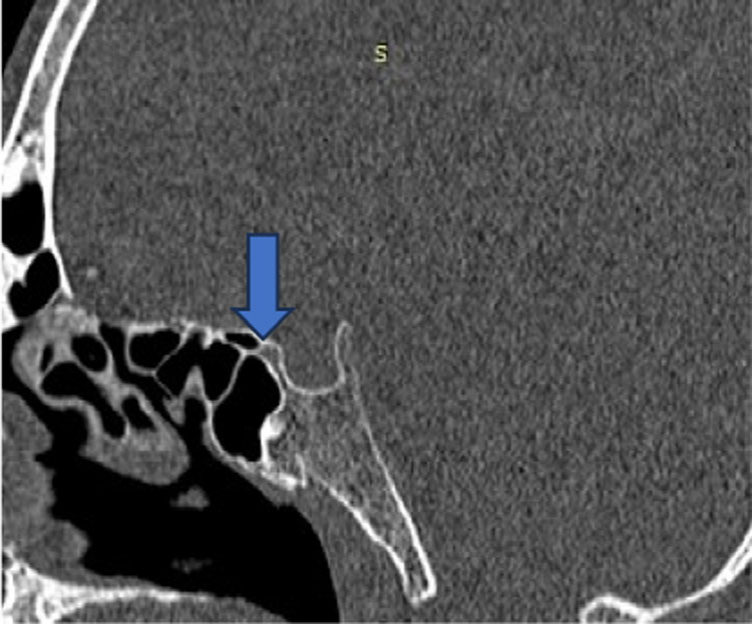
CT Sagittal image shows presellar pneumatization of sphenoid sinus.

**Figure 5:**
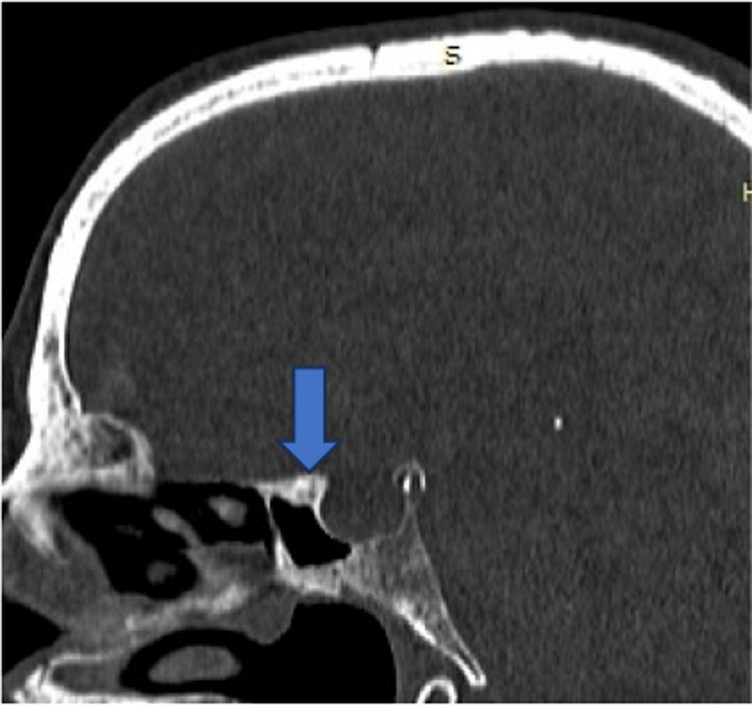
CT Sagittal image shows sellar pneumatization of sphenoid sinus.

**Figure 6:**
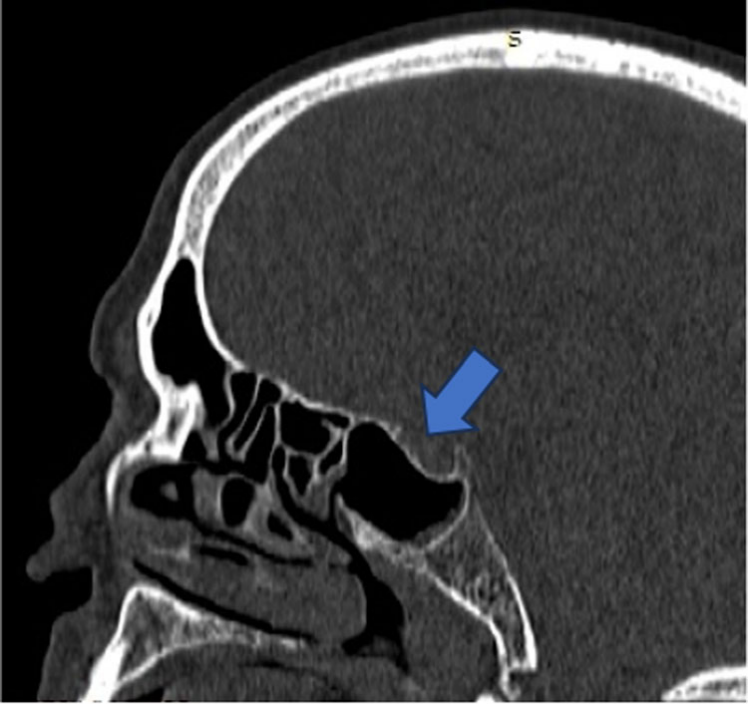
CT Sagittal image shows post‐sellar pneumatization of sphenoid sinus.

Table 3 shows that out of 153 patients, *n* = 136 (88.9%) don't have presellar sphenoid sinus with respect to sella turcia, but *n* = 17 (11.1%) have presellar sphenoid sinus (Figure 4), *n* = 110 (71.9%) don't have sellar sphenoid sinus with respect to sella turcia, and *n* = 43 (28.1%) have sellar sphenoid sinus (Figure 5), *n* = 66 (43.1%) don't have postsellar sphenoid sinus with respect to sella turcia, while *n* = 87 (56.9%) have postsellar sphenoid sinus (Figure 6), *n* = 150 (98.0%) don't have conchal sphenoid sinus with respect to sella turcia, and *n* = 3 (2.0%) have conchal sphenoid sinus.

**Table 3:** Anatomical variations of sphenoid sinus.

Sinus Type	Normal variations	Occurance	*N*
Sphenoid sinus	Conchal	Present 3 (2.0%)	153
		Absent 150 (98.0%)	
	Presellar	Present 17 (11.1%)	
		Absent 136 (88.9%)	
	Sellar	Present 43 (28.1%)	
		Absent 110 (71.9%)	
	Postsellar	Present 87 (56.9%)	
		Absent 66 (43.1%)	

**Figure 7:**
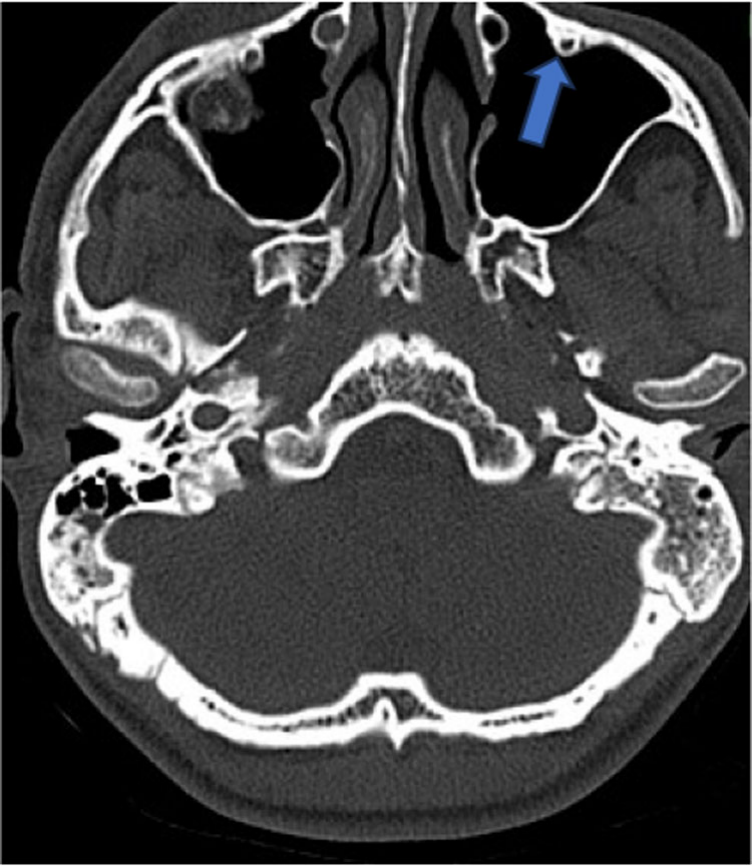
CT axial image shows exostosis of maxillary sinus.

**Figure 8:**
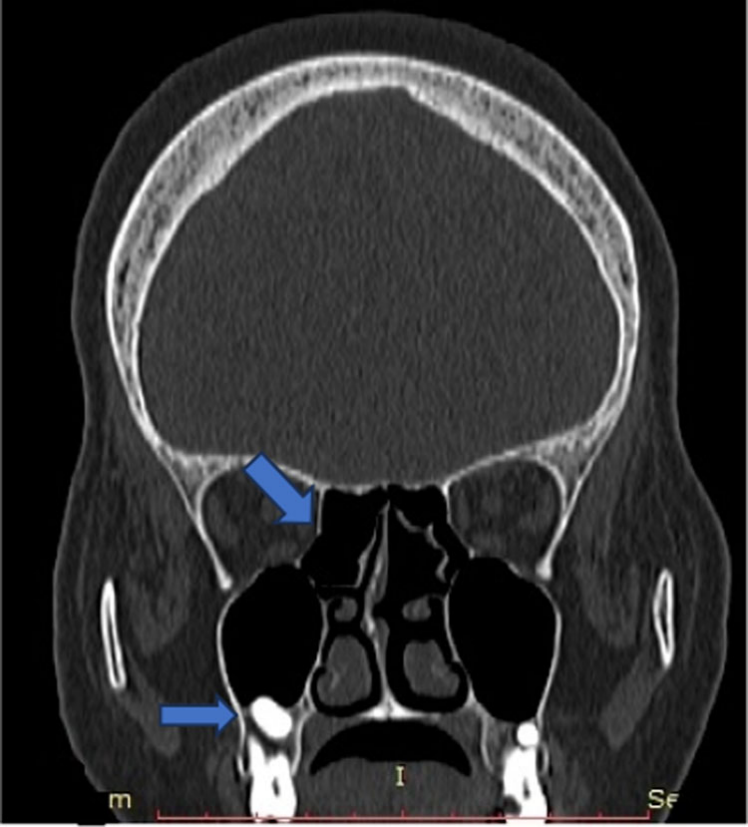
CT coronal image shows extension of maxillary sinus to teeth roots, in ethmoid sinus ethoidal bulla is seen.

**Figure 9:**
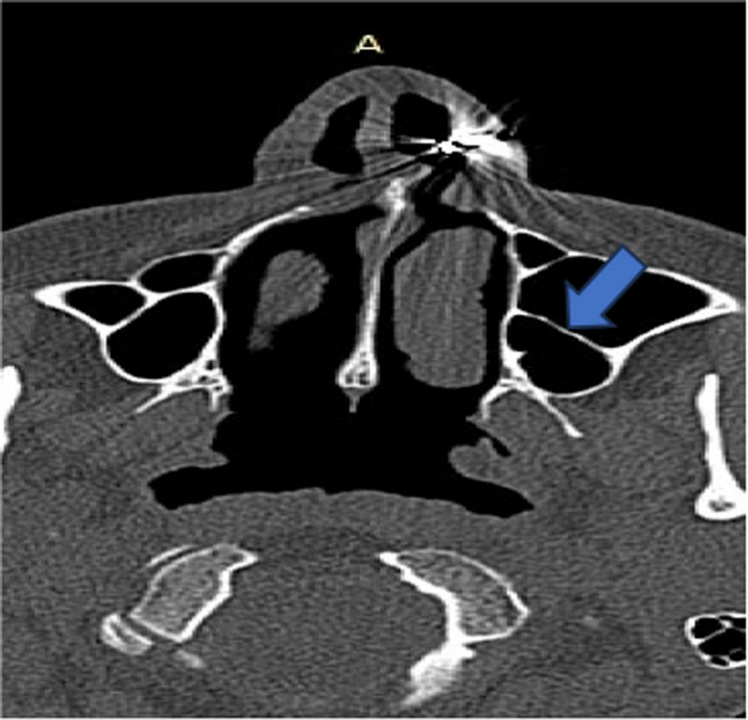
CT axial image shows septations of maxillary sinus.

Table 4 shows that out of 153, *n* = 152 (99.3%) have no hypoplasia of the maxillary sinus and 1 (0.7%) has hypoplasia of the maxillary sinus, *n* = 139 (90.8%) have no exostosis of the maxillary sinus, but *n* = 14 (9.2%) have exostosis of the maxillary sinus (Figure 7), *n* = 132 (86.3%) have no extension of teeth roots to the maxillary sinus, but *n* = 21 (13.7%) have extension of teeth roots to the maxillary sinus (Figure 8), and *n* = 148 (96.7%) have no septations in the maxillary sinus, but *n* = 5 (3.3%) have septations in the maxillary sinus (Figure 9).

**Table 4:** Anatomical variations of maxillary sinus.

Sinus Type	Normal variations	Occurance	*N*
Maxillary sinus	Hypoplasia	Present 1 (0.7%)	153
		Absent 152 (99.3%)	
	Exostosis	Present 14 (9.3%)	
		Absent 139 (90.8%)	
	Extension to teeth roots	Present 21 (13.7%)	
		Absent 132 (86.3%)	
	Septations	Present 5 (3.3%)	
		Absent 148 (96.7%)	

**Figure 10:**
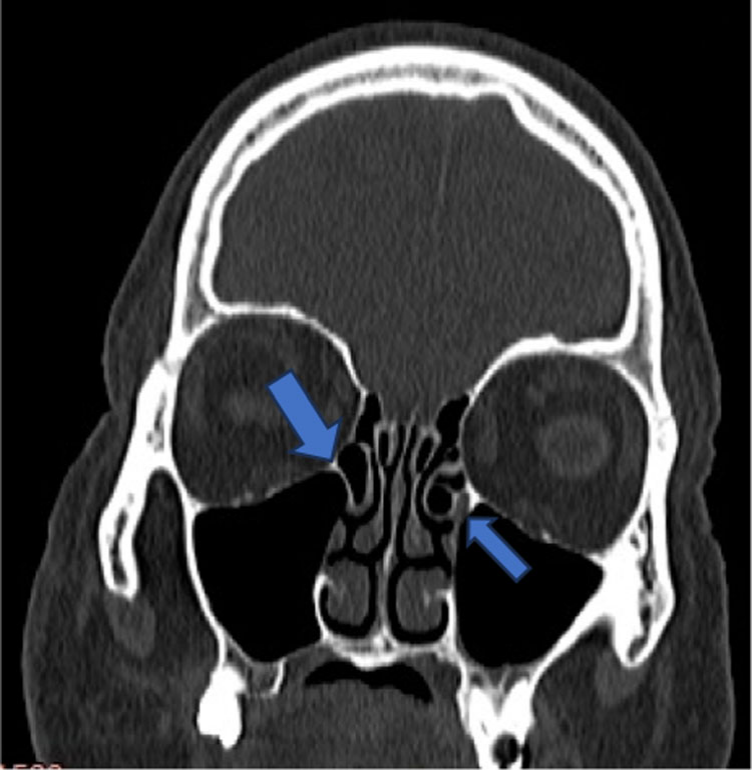
CT coronal image shows ethmoidal bulla or haller cells.

**Figure 11:**
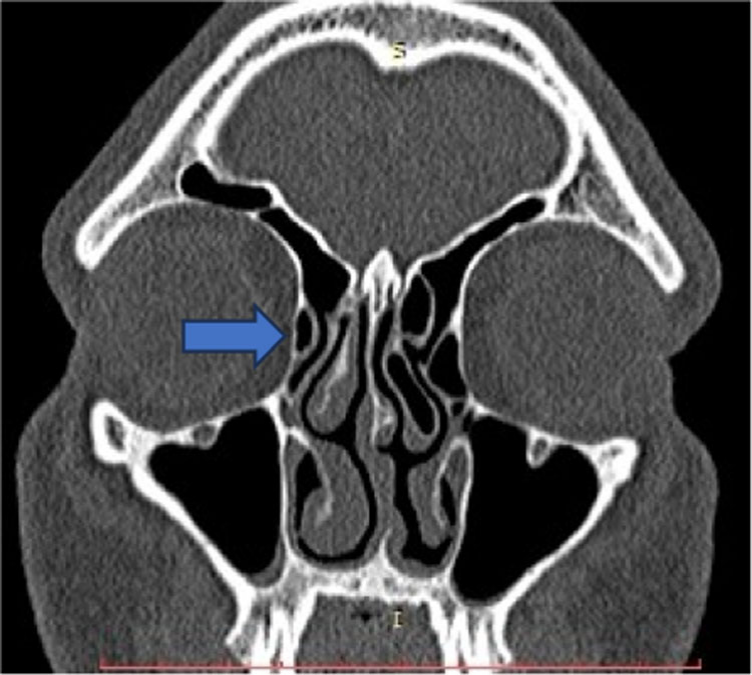
CT coronal image shows agger nasi cell in ethmoid sinus.

Table 5 shows that out of 153, *n* = 78 (51%) have no ethmoidal bulla in the ethmoid sinus, but *n* = 75 (49%) have ethmoidal bulla (Figure 10); *n* = 103 (67.3%) have no agger nasi cells in the ethmoid sinus, while *n* = 50 (32.7%) have agger nasi cells (Figure 11); and *n* = 106 (69.3%) don't have haller cells in the ethmoid sinus, but *n* = 47 (30.7%) have haller cells.

**Table 5:** Anatomical variations of ethmoid sinus.

Sinus type	Normal variations	Occurance	*N*
Ethmoid sinus	Ethmoidal bulla	Present 75 (49.0%)	153
		Absent 78 (51.0%)	
	Agger nasi cells	Present 50 (32.7%)	
		Absent 103 (67.3%)	
	Haller cells	Present 47 (30.7%)	
		Absent 106 (69.3%)	

## 4 Discussion

In this study, by using MDCT, we aimed to comprehensively evaluate the frequency of the normal anatomical variations of paranasal sinuses in a large population. The results of our research reveal a high prevalence of anatomical variations in bilateral frontal sinuses, that is, more than two chambers, post‐sellar pneumatization of the sphenoid sinus, ethmoidal bulla in the ethmoid sinus, and extension of teeth roots to the maxillary sinus. However, such characteristics and findings from our study also provide a new perspective on the frequency and co‐occurrence of these normal anatomical variations of paranasal sinuses by highlighting the importance of individualized assessment and consideration of these variations in both diagnostic and therapeutic decision‐making.

A study by Amit Nautiyal et al. (2017) found that the frequency of Haller cells (22.9%) and agger nasi cells (21.6%) is somewhat similar to our findings [20]. Mohammed et al. (2019) carried out a hospital‐based cross‐sectional research. The findings of this study showed that among 392 participants, the absolute frequency of anatomical changes was 51.0% for the agger nasi cell and 30.1% for the Haller air cell. In 29.9% of cases, the significant ethmoidal bulla was seen [18]. These prevalence rates may vary depending on the ethnic makeup of the communities and the methods used. These prevalence rates may differ because of the methods used and the sample size.

The findings of our investigation into variations in the frontal sinus include aplasia at 4.6%, hypoplasia with a metopic suture at 9.8%, bilateral aplasia at 84.3%, and unilateral aplasia at 13.7%. In a previous study conducted by Aydinlioğlu et al., the overall frequency of a bilateral absence of the frontal sinus was 3.8%; Males make up 1.3% and females make up 5.1% [21]. Rajendra Kumar Narasipur Lingaiah et al. performed a study in 2016 in which the researchers found that the frontal sinus septations were present in 31% of patients, which concluded that in most of the patients bilateral or more than two chambers were present [14]. Mallikarjun M. Devareddy and Shilpa Devakar conducted a study in 2019; the recorded frontal sinus septations were found in 26% of patients [19].

Our study of sphenoid sinus variations revealed that the majority of the sinuses had a post‐sellar pattern of pneumatization, with conchal 2%, presellar 11.1%, sellar 28.1%, and postsellar 56.9%. A prospective study was carried out in 2017 at the Tikur Anbessa Specialized Teaching Hospital, Addis Ababa University, which revealed that the anatomic alternates of the sphenoid sinus were 100 (50%) seller, 51 (25.5%) presellar, 45 (22.5%) postsellar, and 4 (2%) conchal types of pneumatization [22]. Bursa Pirinc et al. carried out a study in 2019 using MDCT to evaluate the paranasal sinuses in 200 individuals. The type of sphenoid sinus was found to have a significant impact on the volume averages. The sellar type of sphenoid sinus was seen the most frequently (41.5%), followed by the postsellar type (38.5%), and the presellar type was seen the least frequently (9%). Even though there are no pituitary adenomas in the sphenoid sinus, its volume is larger in males than in females. The sphenoid sinus continues to grow until the age of nine [23].

This study on maxillary sinus variations revealed hypoplasia of 0.7%, exostoses of 9.2%, extension to the roots of the teeth of 13.7%, and septations of 3.3%. In 2016, a study by Meryem Toraman Alkurt et al. found that exostoses were more common in females (58.3%) than in males (41.7%) [24]. Talo Yildirim et al. (2017) found that seventy maxillary sinus septa, 26.1%, were found to have a mediolateral orientation. There were no significant gender or crest type differences in any aspect of the maxillary sinus septa [25]. A study conducted by Demet Yazici et al. (2018) found maxillary sinus hypoplasia in 6 (2.6%) patients [12]. Another study by Mallikarjun M. Devareddy and Shilpa Devakar (2019) found maxillary sinus septa in 15% of patients [19].

The data in Table 6 serve to compare the findings of the present study with previous research on anatomical variations of the paranasal sinuses, highlighting both similarities and distinctions. For example, the frequency of frontal sinus aplasia observed in this study (4.6%) aligns closely with findings by Earwaker (5%) and Alshaikh (7.3%), providing consistency in the understanding of this variation across different populations. In terms of sphenoid sinus pneumatization, the postsellar type was predominant in this study at 56.9%, which is notably higher than in other studies, suggesting a distinct pattern in this anatomical feature within the sampled population. Additionally, the occurrence of maxillary sinus septations (3.3%) is lower than reported by Midilli et al., though it closely matches Yazici's findings, indicating that this variation may have a relatively low but consistent prevalence. This comprehensive comparative analysis reinforces the importance of understanding these variations for clinical and surgical planning. Sinus surgeons must have a thorough understanding of these anatomic variations before surgery to avoid devastating complications. By providing the anatomical “road map” necessary to identify the presence of all anatomical variations with the increased clarity and accuracy required before any surgical access, CT plays a crucial role.

**Table 6:** Comprehensive comparative analysis of anatomical variations of paranasal sinuses between current study and previous studies.

Anatomical variant of paranasal sinus	Percentage (%) variant								
	Current study	Alshaikh and Aldhurais [26]	Earwaker [27]	Midilli et al. [28]	Perez‐Pinas et al. [29]	Nautiyal et al. [20]	Onwuchekwa and Alazigha [2]	Devareddy MM et al. [19]	Yazici D. [12]
**Frontal sinus**									
Aplasia	4.6	7.3	5.0	4.2	—	—	—	—	3.1
Unilateral (bilateral)	13.7 (84.3)	—	—	—	—	—	—	—	—
More than two chambers	42.5	—	—	—	—	—	—	—	—
Hypoplasia with persistent metopic suture	9.8	26.9	4.0	14.1	—	4.64	3.64	—	2.6
**Sphenoid sinus**									
Conchal	2.0	44.3	55.0	37.0	—	—	—	—	—
Presellar	11.1	—	—	—	—	—	—	—	—
Sellar	28.1	—	—	—	—	—	—	—	—
Postsellar	56.9	—	—	—	—	—	—	—	—
**Maxillary sinus**									
Hypoplasia	0.7	5.0	—	4.0	6.3	0.91	0.91	1.0	2.6
Exostosis	9.3	—	—	—	—	—	—	—	—
Extension to teeth roots	13.7	—	—	—	—	3.73	2.73	—	—
Septations	3.3	8.6	—	2.0	—	7.36	6.36	5.3	—
**Ethmoid sinus**									
Ethmoidal bulla	49.0	—	—	—	—	—	—	—	—
Agger nasi cells	32.7	100	96.0	—	85	21.64	23.64	9.2	85.3
Haller cells	30.7	32.4	21.0	—	3.0	22.91	20.91	5.6	20.9

## 5 Limitations

The study's limitations include its small sample size and single‐center approach, which limit the generalizability of findings to broader populations. As a cross‐sectional study, it provides only a snapshot without longitudinal insights. Additionally, there was no comparison between MDCT findings and FESS outcomes, which could have validated the accuracy of MDCT in surgical planning. The study sample was also demographically homogeneous, possibly limiting applicability to diverse populations. Lastly, the use of a SIEMENS 64‐slice CT machine may affect findings, as results could vary with other imaging equipment or newer technologies.

## 6 Conclusion

The most common anatomical variations are bilateral frontal sinus, post seller pneumatization of sphenoid sinus, ethmoidal bulla in ethmoid sinus and extension of teeth roots to maxillary sinus. Knowledge of these anatomical variations preoperatively is of sheer importance for surgeons, and failure to pay attention to these minor details may lead to destructive circumstances. Such studies play an important role in patient care and can lead to a reduction in adverse sequelae of surgical intervention.

## Author Contributions

**Babar Ali:** conceptualization, methodology, data curation, formal analysis, writing – original draft, investigation, supervision, visualization, project administration, writing – review and editing, validation, software, resources. **Zonaina Nadeem:** conceptualization, data curation, writing – review and editing, validation, visualization, resources, methodology, formal analysis. **Muhammad Naeem:** conceptualization, software, investigation, formal analysis, data curation, visualization, project administration, writing – original draft. **Kinza Arif:** conceptualization, methodology, software, data curation, investigation, validation, resources, writing – original draft, writing – review and editing, visualization. **Asad Zama:** investigation, project administration, writing – review and editing, validation, data curation, methodology, writing – original draft, conceptualization. **Aqsa Noor:** conceptualization, investigation, formal analysis, writing – original draft, data curation, resources, validation, software. **Muhammad Hassaan Zahid:** methodology, software, validation, formal analysis, visualization, resources, data curation, conceptualization, project administration, writing – review and editing. **Rimsha Mustafa:** conceptualization, formal analysis, software, validation, resources, methodology, visualization, project administration, writing – review and editing. **Aymar Akilimali:** Project administration, visualization, validation, software, investigation, resources, funding acquisition, writing ‐ review and editing, formal analysis. **Jacob Leonard Ago:** data curation, investigation, visualization, writing ‐ review and editing, software.

## Conflicts of Interest

The authors declare no conflicts of interest.

## Transparency Statement

The lead author Aymar Akilimali affirms that this manuscript is an honest, accurate, and transparent account of the study being reported; that no important aspects of the study have been omitted; and that any discrepancies from the study as planned (and, if relevant, registered) have been explained.

## Data Availability

The data that support the findings of this study are available on request from the corresponding author. The data are not publicly available due to privacy or ethical restrictions.
